# Associations of leucocyte telomere length with cardio-metabolic risk profile in a South African HIV-infected population

**DOI:** 10.1097/MD.0000000000028642

**Published:** 2022-02-04

**Authors:** Ndonwi Elvis Ngwa, Nasheeta Peer, Tandi E. Matsha, Anniza de Villiers, Eugene Sobngwi, Andre P. Kengne

**Affiliations:** a South African Medical Research Council/Cape Peninsula University of Technology Cardio-metabolic Health Research Unit, Department of Biomedical Sciences, Faculty of Health and Wellness Sciences, Cape Peninsula University of Technology, Cape Town, South Africa; b Non-Communicable Diseases Research Unit, South African Medical Research Council, Cape Town and Durban, South Africa; c Department of Medicine, Faculty of Health Sciences, University of Cape Town, South Africa; d Laboratory for Molecular Medicine and Metabolism, Biotechnology Center, University of Yaoundé 1, Yaoundé, Cameroon.

**Keywords:** Africa, cardiometabolic risk factors, HIV, hypertension, leukocyte telomere length, obesity

## Abstract

Leukocyte Telomere length (LTL) is an independent predictor of cardio-metabolic diseases (CMDs) and Human Immuno Virus (HIV) infection. However, studies are lacking on the association between LTL with CMD profile in people with HIV. Accordingly, we investigated the association between LTL and CMD profile in HIV-infected adult South Africans.

This cross-sectional study included 728 HIV patients (20.6% men; median age 38 years) recruited across 17 public healthcare facilities in Cape Town. CMD markers were compared across quartiles of LTL, and spearman correlations assessed the continuous association of LTL with CMD markers. Linear and logistic regressions were then used to relate LTL with CMD risk profile, with appropriate adjustment for confounders.

The prevalence of obesity, hypertension and diabetes were 34.8%, 36.8%, and 8.4%, respectively. In age, sex and body mass index adjusted models, increasing Log_10_LTL was associated with decreasing systolic (β = −10.52) and diastolic (β = −6.74) blood pressures, HOMA-β (β = −70.72), increasing total cholesterol (β = 0.544), non-high-density lipoprotein cholesterol (β = 0.472), and waist-to-height-ratio > 0.5 (odds ratio [OR] = 5.67), all *P* < .05. Compared to those in the bottom quarter, those in the top LTL quarter had lower prevalence of hypertension (OR = 0.65), and higher prevalence of total cholesterol > 5 mmol/L (OR = 1.94), and low-density lipoprotein-cholesterol > 3 mmol/L (OR = 1.62), all *P* < .05. LTL was not associated with diabetes nor general obesity. It was associated with Alanine Transaminase (ALT) and heart rate in univariable analyses.

LTL shortening was associated with some CMD risk factors in HIV-infected adults on anti-retroviral therapy in South Africa. Prospective research is needed to explore the direction and implications of these associations.

## 1 Introduction

Telomere is a region of repetitive nucleotide sequences at each end of a chromosome, which protects the end of the chromosome from deterioration or from fusion with neighboring chromosomes.^[1]^ Another important function of telomere involves enabling linear deoxyribonucleic acid (DNA) to replicate completely.^[1]^ Therefore, in the absence of telomere, important genes would be deleted during each cell division, negatively affecting the process of DNA replication which is essential for life. During each cell division, a small portion of telomeric DNA is lost resulting in a decline in telomere length (TL)^[1]^ indicating that TL decreases with age.

In addition to this natural process, the decrease in TL can be exacerbated by infectious and non-infectious diseases. Consistent findings have reported associations between short TL and numerous conditions such as HIV infection,^[2,3]^ stroke,^[4]^ ischaemic heart disease,^[5]^ type 2 diabetes mellitus (hereafter referred to as diabetes),^[6]^ and hypertension.^[7,8]^ However, little is known about the relationship of TL with cardio-metabolic diseases (CMDs) in the HIV-infected population in South Africa, the country with the greatest number of people living with HIV (PLWH) globally. The estimated number of HIV cases in the South African population was ∼7.97 million in 2019.^[9]^ South Africa has the largest antiretroviral therapy (ART) programme worldwide with 4.8 million PLWH receiving ART in 2018.^[10]^ Consequently, early morbidity and mortality associated with HIV infection has declined following the widespread uptake of ART and, with longevity, PLWH are now exposed to non-communicable diseases, particularly CMD. The development of CMD in PLWH is linked to ageing and exposures to the traditional lifestyle/behavioural risk factors as in general populations^[11–13]^ together with chronic inflammation caused by HIV infection and side effects of ART.^[14–16]^ This is important in the South African context where, diabetes, cardiovascular diseases, and HIV infection are the 2nd, 4th, and 5th highest contributors to mortality in the country, respectively.^[17]^

In view of the converging burdens of HIV infection and CMD in a large proportion of the South African population, it is important to examine the impact of these comorbidities on health status. Considering that TL is a good indicator of biological age vs chronological age and reflects an individual's health status, examining differences in TL in PLWH with and without CMD provides a unique opportunity to evaluate these associations. Therefore, this cross-sectional study aimed to investigate the association of LTL with CMD risk profile in a sample of adult South Africans living with HIV infection.

## 2 Methods

### 2.1 Study design and population

In this cross-sectional study, HIV-infected participants ≥18 years of age and receiving ART were recruited from primary health care facilities in the Western Cape province of South Africa between March 2014 and February 2015. Sixty-two health facilities, 42 in Cape Town and the others from the surrounding rural municipalities, that provided ART to at-least 325 patients per month, were included. Amongst these 62 health facilities, 17 were randomly selected (4 rural) with 15 to 60 participants recruited from each facility.

The South African Medical Research Council Ethics Committee approved the study, which was conducted in accordance with the principles of the Declaration of Helsinki. The Health Research Office of the Western Cape Department of Health, and the selected healthcare facilities granted permission for participants to be recruited from the health facilities.

### 2.2 Data collection and sampling

Trained fieldworkers administered survey questionnaires, while clinical measurements and biochemical analyses were performed by clinicians and nurses. On the day of recruitment, socio-demographic information, physical assessments (anthropometry and blood pressure) and medical history of HIV infection (duration of diagnosed HIV infection, CD4 counts, and HIV treatment) were collected using a structured interviewer-administered questionnaire adapted from the WHO STEP-wise approach to Surveillance (STEPS) tool. Socio-demographic information was self-reported while medical history of HIV was obtained from medical records and by capturing medications brought to the clinics by participants.

Height, weight, waist circumference (WC), and hip circumference (HC) were measured in duplicate using standardised techniques.^[18]^ Height to the nearest 0.1 cm and weight to the nearest 0.1 kg were taken using a Leicester Height Scale and A&D Personal Scaler, respectively, with the participants in light clothing and bare feet. WC and HC were measured to the nearest millimetre using a non-stretchable measuring tape. For each variable, the average of both measurements was used for analysis. Three blood pressure (BP) measurements were taken at 3-minute intervals using a digital automatic BP monitor after the participant had rested for at-least 5 minutes. The average of the second and third BP measurements was used for analysis.

Blood samples were collected the following day in Ethylene Di-amine Tetra Acetate (EDTA) and dry tubes after participants had fasted for at least 8 hours. Serum and plasma were prepared from blood in dry tubes and a portion of blood in EDTA tubes and stored at −20°C for biochemical analyses. The other portion in the EDTA tube (5 mL) was stored at −80°C for LTL measurements. Biochemical parameters were measured at an ISO 15189 accredited pathology laboratory (PathCare, Reference Laboratory, Cape Town, South Africa) which had no access to participants’ clinical information. Plasma glucose (hexokinase), serum creatinine (cayman chemical), gamma glutamyl transferase (GGT) (Abcam) were measured using colorimetric methods according to the manufacturer's protocol. Estimated Glomerular Filtration rate (eGFR) was calculated using the Modification of Diet in Renal Disease Study (MDRD) equation; eGFR = 175 × Creatinine (mg/dL)^−1.154^ × Age (years)^−0.203^ × 0.742 (female).^[19]^ Total cholesterol (TC), high-density lipoprotein cholesterol (HDL-C) and triglycerides were measured in serum using standard enzymatic techniques.^[20–22]^ Low-density lipoprotein cholesterol (LDL-C) was calculated using the Friedewald equation,^[23]^ while non-HDL-C was calculated using the formula: TC – HDL-C.

Plasma alanine transaminase (ALT) and aspartate transaminase (AST) levels were measured with ALT and AST reagent kits, respectively, according to the manufacturer's protocol (Thermo-Fisher Scientific). All colorimetric measurements were carried out using Beckman Coulter AU 500 spectrophotometer. Plasma insulin concentrations were measured by the Chemiluminescence Immunoassay method (Human insulin CLIA kit: Abnova), glycated haemoglobin (HbA1c) levels were determined using high-performance liquid chromatography technique (Thermo-Fisher Scientific) and highly sensitive c-reactive protein (hs-CRP) measured by Enzyme Linked Immuno-Sorbent Assay (ELISA) method (BIOMATIK) according to the manufacturer's protocol.

For LTL assay, DNA was extracted from blood samples using the salt extraction technique. Briefly, 5 mL blood samples in EDTA tubes were defrosted to 4°C and poured into a 50 mL centrifuge tube. Thirty mL lysis buffer was added, and red blood cells were lysed by incubation on ice and vortexing. After lysis of red blood cells, the pellets were washed thrice with phosphate buffered saline which was later discarded. The pellets were then incubated with nuclear lysis buffer overnight at 60°C. The next day, the supernatant was collected, and the proteins precipitated using 1 mL saturated sodium chloride (6 M) solution. The supernatant containing the DNA was collected into new 15 mL centrifuge tubes and absolute ethanol added to precipitate the DNA by inversion. Precipitated DNA was removed and washed with 70% ethanol. After washing, the precipitate was dissolved in Tris-EDTA (TE) buffer and the concentration and quality of the DNA measured using a Nano drop. All samples with optical density from 1.7 to 2 were diluted to 5 mg/mL using polymerase chain reaction (PCR) grade water and TL measured by quantitative real time polymerase chain reaction (PCR) using the method described by O’Callaghan and Fenech.^[24]^

Serial dilutions of the telomere standard and the single copy gene (36B4) standard were made. A master mix solution containing Power SYBR I (AmpliTaq Gold DNA polymerase, dNTPs, SYBR I Green Dye, optimised buffers and passive reference dye (ROX) (10 μL, 1×), forward primer (1 μL, 0.1 μM), reverse primer (1 μL, 0.1 μM), ddH_2_O (4 uL) was prepared, mixed well and briefly centrifuged. Using a multichannel pipetted, 16 μL master mix solution were pipetted into each well of a 96 well plate. Into the corresponding wells were added 4 μL each of DNA sample, standards, positive and non-template control (distilled water). The plate was sealed with an optical clear film, centrifuged briefly and run using the following PCR conditions; 10 minutes at 95°C, followed by 40 cycles of 95°C for 15 seconds 60°C for 1 minute, followed by a dissociation (or melt) curve. At the end of the run, the plate was removed and discarded. Each sample was amplified twice, using telomere forward and reverse primers and the single copy gene forward and reverse primers. After amplification is completed the AB software produces a value for each reaction that is equivalent to kb/reaction based on the telomere standard curve values. The kb/reaction for telomere and genome copies/reaction for diploid genome copy values are exported as used to calculate the LTL as follows; $\text{LTL}=\frac{\text{telomere}\;\text{kilobase}\;\text{per}\;\text{reaction}\;\text{value}}{\text{diploid genome copy number}}$

### 2.3 Definitions

Body mass index (BMI) was calculated as weight (kg)/height (m) squared, and participants classified into three categories of generalised adiposity: normal weight (BMI <25 kg/m^2^), overweight (BMI ≥25 kg/m^2^ and BMI <30 kg/m^2^) and obese (BMI ≥30 kg/m^2^).^[25]^ Central obesity was determined using the following criteria:

1. WC > 94 cm in men and >80 cm in women or
2. waist-to-hip ratio (WHR) ≥ 0.9 in men and ≥ 0.85 in women^[26]^ or
3. waist-to-height ratio (WHtR) > 0.5.^[27]^

Hypertension was defined as systolic BP (SBP) ≥140 mm Hg and/or diastolic BP (DBP) ≥90 mm Hg or known hypertension on treatment.^[28]^ Dyslipidaemia was defined as TC > 5 mmol/L, triglycerides >1.5 mmol/L, HDL-C < 1.2 mmol/L, LDL-C > 3.0 mmol/L, and non-HDL-C > 3.37 mmol/L or ongoing lipid control agents.^[29]^ Diabetes was defined as fasting plasma glucose ≥7.0 mmol/L and/ or 2-hour post glucose load ≥11.1 mmol/L or participants taking antidiabetic medications.^[30]^

Insulin resistance (IR) was based on the homeostasis model assessment (HOMA) using the formula: $\text{HOMA-IR}=\frac{\text{Fasting}\;\text{Glucose}(\text{nmol}/\text{L})\times \text{Fasting}\;\text{Insulin}(\text{microU}/\text{L})}{22.5}$, Beta cell function was determined by HOMA-β using the formula: $\text{HOMA-}\beta =\frac{20\times (\text{Fasting}\;\text{Insulin}(\mu \text{IU}/\text{mL})}{\text{Fasting}\;\text{Glucose}(\text{mmol}/\text{mL})-3.5)}$.^[31]^

### 2.4 Statistical analysis

Participants’ characteristics were summarized as medians (25th–75th percentiles) for continuous variables and as counts (percentages) for categorical variables. Comparison of baseline characteristics by sex was carried out using Mann–Whitney *U* test. TL was categorised into four quartiles and the linear trend in CMD profile (continuous variables) across the different quartiles of TL was computed using the median test. Similarly, chi square test was computed and the Cochrane trend test was used to assess the linear trend in proportions across the quartiles of TL. Spearman correlation was used to assess the association between TL and cardio-metabolic parameters. Linear and logistic regressions were then used to investigate the independent association of TL with CMD risk profile, while accounting for the possible effects of confounders. All analyses were carried out using statistical packages for social science (SPSS) Version 21.

## 3 Results

### 3.1 Clinical characteristic of the study sample

The clinical characteristics of the study participants are shown in Table 1. Amongst the 833 participants included in the study, TL data were available for 728 of whom 93% provided information on ART. The participants’ average age was 36 years with men significantly older than women (41 years vs 37 years, *P* < .001). The median CD4 count was 400 cells/mm^3^ with counts significantly higher in women (410 cells/mm^3^) compared to men (272 cells/mm^3^), *P* = .001. However, LTL was not significantly different between men and women (*P* = .214). Amongst the participants receiving ART (n = 675), the majority were receiving 1st line ART (56.3%) with the distribution of ART regimens differing in men and women (*P* = .005). All measures of obesity were significantly higher in women compared with men (*P* < .001). However, the prevalence of hypertension (36.8%) and diabetes (8.4%) were similar in men and women. Among participants with hypertension (n = 268) and diabetes (n = 61), the majority (60.8% and 54.1%, respectively) were newly diagnosed.

**Table 1:** Clinical characteristics of the study population.

Gender	N	Total	Male	Female	*P*
Number (%)		728	150 (20.6)	578 (79.4)	
Median values (25th–75th percentile)					
TL (kilobase)	728	77.8 (64.7–95.0)	73.7 (62.0–98.5)	78.2 (65.5–94.7)	.214
Age (yr)	726	38.0 (32.0–45.0)	41.0 (35.0–47.0)	37.0.3 (31.0– 44.0)	<.001
Weight (kg)	725	68.5 (58.2–82.0)	62.4 (55.3–71.2)	71.3 (58.9–84.7)	<.001
Height (cm)	725	160.6 (156.0–166.2)	170.5 (165.1–173.5)	159.0 (155.0–163.1	<.001
BMI (kg/m^2^)	725	26.4 (22.1–32.3)	21.4 (19.8–24.2)	28.4 (23.8–33.5)	<.001
Waist Circumference (cm)	725	88.0 (77.6–98.2)	79.0 (74.3–88.4)	90.3 (79.2–101.2)	<.001
Hip Circumference (cm)	725	102.2 (92.7–112.2)	91.8 (88.1–97.1)	105.2 (96.8–116.1)	<.001
WHR	725	0.86 (0.80–0.91)	0.87 (0.83–0.92)	0.85 (0.80–0.90)	<.001
WHtR	725	0.55 (0.48–0.61)	0.47 (0.44–0.52)	0.57 (0.50–0.64)	<.001
SBP (mm Hg)	725	117.0 (107.0–129.5)	123.5 (114.5–139.9)	115.0 (106.0–127.0)	<.001
DBP (mm Hg)	725	82.0 (75.0–90.5)	82.5 (76.0–92.8)	82.0 (74.5–90.0)	.225
Heart rate (beats/min)	725	75.0 (67.0–83.0)	69.5 (61.3–81.8)	75.0 (68.0–83)	<.001
CD4 count (cells/mm^3^)	371	400.0 (241–608.0)	282.0 (180.5–457.5)	417.0 (256.8–630.8)	.001
ALT (IU/L)	712	23.0 (17.0–34.0)	25.5 (19.0–42.0)	22.0 (16.0–32.0)	<.001
AST (IU/L)	711	29.0 (24.0–38.0)	34.5 (27.0–45.0)	28.0 (23.0–35.0)	<.001
Total cholesterol (mmol/L)	711	4.3 (3.7–5.1)	4.2 (3.5–5.0)	4.5 (3.8–5.1)	.018
HDL-C (mmol/L)	711	1.3 (1.0–1.5)	1.2 (1.0–1.5)	1.3 (1.1–1.5)	.017
Triglycerides (mmol/L)	710	1.0 (0.8–1.4)	1.1 (0.8–1.6)	1.0 (0.7–1.3)	.014
LDL-C (mmol/L)	711	2.5 (2.0–3.1)	2.3 (1.9–3.0)	2.5 (2–3.2)	.021
Non-HDL-C (mmol/L)	665	3.0 (2.5–3.7)	3.0 (2.3–3.6)	3.0 (2.5–3.7)	.151
Creatinine (μmol/L)	710	58.0 (51.0–67.0)	70.0 (61.0–79.0)	56.0 (50.0–62.0)	<.001
hs-CRP (mg/L)	711	5.4 (2.4–14.0)	4.6 (1.8–14.5)	5.6 (2.4–13.9)	.391
GGT (U/L)	711	39.0 (26.0–66.0)	52.0 (30.0–95.0)	38.0 (25.0–58.0)	<.001
Fasting glucose (mmol/L)	711	5.0 (4.6–5.4)	5.1 (4.8–5.5)	4.9 (4.6–5.4)	.005
2 h glucose (mmol/L)	645	5.30 (4.59–6.20)	5.20 (4.40–6.30)	5.40 (4.59–6.15)	.405
Insulin (mU/L)	679	6.1 (4.0–9.6)	4.0 (2.5–6.4)	6.6 (4.4–10.3)	<.001
HOMA-IR	678	1.4 (0.9–2.3)	0.93 (0.52–1.71)	1.5 (1.0–2.4)	<.001
HOMA-β	671	82.1 (50.0–131.1)	51.2 (32.3–72.9)	92.2 (56.9–142.2)	<.001
HbA1c (%)	712	5.4 (5.2–5.7)	5.5 (5.3–5.8)	5.4 (5.2–5.7)	.203
eGFR (mL/min)	710	106.1 (93.1– 123.8)	109.0 (94.3–127.1)	105.1 (93.0–123.2)	.107
Prevalence, % (n)					
Adiposity					
BMI ≥ 30 kg/m^2^	722	34.6 (246)	6.8 (10)	41.9 (236)	<.001
WC: men >94 cm, women >80 cm	722	61.8 (448)	15.5 (23)	73.7 (425)	<.001
WHR: men ≥ 0.9, women ≥0.85	722	48.7 (353)	33.1 (49)	52.7 (304)	<.001
WHtR > 0.5	722	65.1 (472)	31.8 (47)	73.7.6 (425)	<.001
Hypertension	726	36.8 (268)	38.5 (57)	36.6 (211)	.654
Diabetes	726	8.4 (61)	11.4 (15)	8.9 (46)	.433
Dyslipidaemia					
TC > 5.0 mmol/L	711	25.5 (181)	23.6 (35)	25.9 (146)	.571
HDL-C < 1.2 mmol/L	711	37.3 (265)	46.6 (69)	34.8 (196)	.008
Triglyceride > 1.5 mmol/L	710	18.2 (129)	28.4 (42)	15.5 (87)	<.001
LDL-C > 3.0 mmol/L	711	28.0 (199)	23.0 (34)	29.3 (165)	.127
Non HDL-C > 3.37 mmol/L	711	33.4 (243)	33.6 (47)	37.3 (196)	.412

ALT = alanine transaminase, AST = aspartate transaminase, BMI = body mass index, DBP = diastolic blood pressure, eGFR = estimated glomerular filtration rate, GGT = gamma glutamyl transferase, HbA1c = glycated haemoglobin, HDL-C = high-density lipoprotein cholesterol, HOMA-β = homeostasis model assessment of beta cell dysfunction, HOMA-IR = homeostasis model assessment of insulin resistance, hs-CRP = highly sensitive c-reactive protein, LDL-C = low-density lipoprotein cholesterol, SBP = systolic blood pressure, TC = Total cholesterol, TL(kb) = Telomere length in Kilobase, WHR = waist-to-hip ratio, WHtR = waist-to-height ratio.

### 3.2 Clinical characteristics across TL quartiles

TL was categorised into four quartiles with the first quartile being the shortest and the 4th quartile the longest. SBP, DBP, hypertension prevalence, and HOMA-β decreased significantly across increasing quartiles of TL (*P* < .05 for all) (Table 2). Moreover, correlation analysis showed a significant negative correlation between TL and all BP parameters (SBP, DBP, hypertension), ALT and HOMA-β. Surprisingly, heart rate was higher in the two longer TL quartiles compared with the shorter TL quartiles. Lipid profile, markers of liver function (ALT and AST), diabetes, HbA1c, and obesity determined by BMI, WC, WHR, and WHtR across quartiles of TL were not significantly different (Table 2).

**Table 2:** Cardio-metabolic profile presented by telomere length quartiles.

	Telomere length category					
Variable	1st quartile median (25–75)	2nd quartile median (25–75)	3rd quartile median (25–75)	4th quartile median (25–75)	*P* for linear trend	Correlation coefficient (2-tailed significance)
n	181	182	182	181		
Median values (25th–75th percentile)						
Age (yr)	37 (30–43)	37 (32–42)	37.5 (32–44)	39 (31–44.5)	.376	−0.028 (0.458)
Weight (kg)	73.5 (58.4–88.6)	72.3 (60.6–83.3)	67.4 (57.6–76.2)	71.8 (59.0–87.3)	.461	−0.030 (0.417)
Height (cm)	161.0 (155.0–166.3)	159.9 (155.5–167.0)	160.0 (155.5–163.5)	159.9 (155.6–166.4)	.784	−0.008 (0.838)
BMI (kg/m^2^)	28.0 (22.6–34.7)	27.7 (22.9–33.2)	26.5 (23.1–29.6)	27.8 (22.1–32.6)	.440	−0.031 (0.402)
Waist circumference (cm)	90.3 (79.5–102.3)	89.3 (81.0–101.4)	88.5 (77.8–95.1)	88.2 (79.9–104.0)	.496	−0.024 (0.511)
Hip circumference (cm)	103.7 (93.5–119.2)	105.0 (94.9–117.0)	102.6 (94.2–109.3)	105.2 (93.9–114.1)	.461	−0.019 (0.607)
WHR	0.86 (0.81–0.90)	0.86 (0.80–0.91)	0.85 (0.79–0.89)	0.86 (0.80–0.92)	.930	−0.017 (0.643)
WHtR	0.54 (0.47–0.61)	0.57 (0.49–0.62)	0.56 (0.49–0.61)	0.52 (0.46–0.62)	.479	−0.028 (0.454)
SBP (mm Hg)	118.0 (112.0–130.0)	118.0 (103.5–128.5)	117 (107.3–129.0)	114.5 (107.0–124.5)	.024^∗^	−0.105^∗∗^ (0.005)
DBP (mm Hg)	83.8 (77.5–91.0)	82.5 (73.0–89.5)	82.3 (77.5–91.3)	81.5 (74.3–89.0)	.042^∗^	−0.089^∗^ (0.016)
Heart rate (beats/min)	74.0 (64.75–81.25)	73.0 (66.0–83.0)	75.0 (66.0–86.0)	75.0 (68.75–83.0)	.033^∗^	0.069 (0.062)
CD4 count (cells/mm^3^)	366.5 (241.0–504.0)	450.0 (226.0–616.0)	418.0 (254.5–661.5)	343.5 (191.5–486.5)	.576	−0.046 (0.373)
ALT (IU/L)	24.0 (18.0–38.0)	23.0 (18.0–34.0)	23.0 (17.5–31.0)	19.0 (15.0–34.0)	.376	−0.075^∗^ (0.046)
AST (IU/L)	31.0 (25.0–39.0)	27.0 (24.0–38.0)	30.0 (25.5–38.0)	28.0 (21.0–38.5)	.811	−0.056 (0.136)
Total cholesterol (mmol/L)	3.9 (3.5–4.9)	4.7 (3.9–5.2)	4.1 (3.6–5.1)	4.4 (3.8–5.3)	.130	0.054 (0.152)
HDL-C (mmol/L)	1.2 (1.1–1.4)	1.3 (1.1–1.6)	1.3 (1.1–1.6)	1.3 (1.0–1.5)	.595	0.003 (0.931)
Triglycerides (mmol/L)	1.01 (0.77–1.25)	1.02 (0.81–1.40)	0.92 (0.67–1.16)	0.98 (0.76–1.37)	.715	−0.005 (0.903)
LDL-C (mmol/L)	2.25 (1.80–2.90)	2.80 (2.20–3.30)	2.3.0 (1.80–2.85)	2.50 (2.10–3.15)	.242	0.040 (0.285)
Non-HDL-C (mmol/L)	2.76 (2.40–3.60)	3.23 (2.74–3.83)	2.79 (2.27–3.42)	3.04 (2.70–3.72)	.218	0.048 (0.221)
Creatinine (μmol/L)	56.5 (50.0–64.0)	59.0 (54.0–69.0)	58.5 (53.0–64.5)	58.0 (50.0–65.5)	.693	−0.002 (0.960)
hs-CRP (mg/L)	4.46 (1.70–14.7)	4.95 (1.34–12.9)	5.58 (2.10–13.38)	6.48 (2.87–17.35)	.194	0.070 (0.062)
GGT (U/L)	38.0 (27.0–60.0)	42.0 (27.0–71.0)	40.5 (24.5–74.0)	32.0 (22.5–73.5)	.139	0.035 (0.347)
Fasting glucose (mmol/L)	4.8 (4.5–5.2)	5.0 (4.7–5.5)	4.9 (4.7–5.4)	5.0 (4.7–5.3)	.994	0.048 (0.200)
2-h glucose (mmol/L)	5.0 (4.3–5.8)	5.4 (4.8–5.8)	5.3 (4.5–6.1)	5.8 (5.1–6.7)	.474	0.025 (0.481)
Insulin (mU/L)	6.8 (4.1–10.2)	6.3 (4.6–10.4)	5.9 (3.6–8.8)	5.8 (3.8–8.8)	.099	−0.064 (0.096)
HOMA-IR	1.49 (0.86–2.15)	1.39 (1.04–2.37)	1.30 (0.79–1.87)	1.27 (0.88–2.08)	.384	−0.062 (0.109)
HOMA-β	92.8 (57.5–161.7)	91.7 (57.6–137.1)	76.1 (45.4–122.7)	85.2 (53.2–118.6)	.019^∗^	−0.082^∗^ (0.034)
HbA1c (%)	5.4 (5.2–5.7)	5.4 (5.2–5.7)	5.4 (5.2–5.7)	5.5 (5.2–5.8)	.705	0.013 (0.726)
eGFR (mL/min)	107.4 (94.9–124.4	104.7 (93.3–120.6)	105.2 (93.8–122.1)	107.0 (92.1–127.1)	.483	−0.006 (0.868)
Prevalence, %						
BMI ≥ 30 kg/m^2^	23.1 (58)	29.7 (74)	22.8 (59)	24.4 (61)	.863	0.006 (0.866)
WC: men >94 cm, women >80 cm	22.5 (101)	27.9 (125)	27.7 (124)	21.9 (98)	.781	0.010 (0.787)
WHR: men ≥ 0.9, women ≥ 0.85	24.6 (87)	25.3 (89)	25.5 (90)	24.6 (87)	.935	−0.003 (0.935)
WHtR > 0.5	23.9 (113)	28.4 (134)	26.3 (124)	21.4 (101)	.126	0.057 (0.126)
Hypertension	28.0 (75)	27.3 (72)	23.9 (64)	20.8 (57)	.037^∗^	−0.078^∗^ (0.037)
Diabetes	18.1 (11)	29.5 (18)	31.1 (19)	21.3 (13)	.650	−0.017 (0.648)
Dyslipidaemia						
Total cholesterol > 5.0 mmol/L	16.0 (29)	30.4 (55)	27.6 (50)	26.0 (47)	.056	0.072 (0.055)
HDL-C< 1.2 mmol/L	26.5 (70)	21.1 (56)	27.5 (73)	24.9 (66)	.718	−0.014 (0.719)
Triglyceride > 1.5 mmol/L	23.3 (30)	25.6 (33)	24.0 (31)	35.0 (35)	.118	0.025 (0.118)
LDL-C > 3 mmol/L	20.1 (40)	29.7 (59)	23.1 (46)	27.1 (54)	.240	0.044 (0.238)
Non-HDL-C > 3.37 mmol/L	21.0 (51)	29.2 (71)	24.7 (60)	25.1 (61)	.203	−0.050 (0.196)

ALT = alanine transaminase, AST = aspartate transaminase, BMI = body mass index, DBP = diastolic blood pressure, eGFR = estimated glomerular filtration rate, GGT = gamma glutamyl transferase, HbA1c = Glycated Haemoglobin, HDL-C = high-density lipoprotein cholesterol, HOMA-β = homeostasis model assessment of beta cell dysfunction, HOMA-IR = homeostasis model assessment of insulin resistance, hs-CRP = highly sensitive c-reactive protein, LDL-C = low-density lipoprotein cholesterol, SBP = systolic blood pressure, WC = Waist circumference, WHR = waist-to-hip ratio, WHtR = waist-to-height ratio. ^∗∗^ = *P* < .01 ^∗^ = *P* < .05.

### 3.3 Age and sex adjusted regressions of telomere length and cardio-metabolic profile

In age and sex adjusted linear regressions, Log_10_ TL was significantly associated with SBP (beta = −0.087; *P* = .011), DBP (beta = −0.087; *P* = .015), TC (beta = 0.081; *P* = .019), non-HDL-C (beta = 0.081; *P* = .029), and HOMA-β ((beta = −0.091; *P* = .017) (Table 3). This pattern was also apparent across increasing quartiles of TL in linear regressions for SBP, DBP, TC, HOMA-β, LDL-C, and non-HDL-C (Table 4). However, neither quartile of TL nor Log_10_TL were associated with markers of adiposity (WC, HC, WHR, BMI) CD4 count, HbA1c, hs-CRP, GGT, insulin levels, HOMA-IR, and creatinine.

**Table 3:** Linear regression models (coefficients and standard errors) for the associations of Log_10_ telomere length with cardio-metabolic variables.

	Age (years)	Gender (female = reference)	BMI (kg/m^2^)	Log_10_LTL	*R*-squared univariate	*R*-squared with confounders
SBP	0.774^∗∗∗^ (0.075)	−7.427^∗∗∗^ (1.834)	0.195 (0.106)	−10.523^∗^ (4.129)	0.012	0.17
DBP	0.304^∗∗∗^ (0.051)	−2.079 (1.234)	0.238^∗∗^ (0.071)	−6.737^∗^ (2.776)	0.011	0.077
TC	0.026^∗∗∗^ (0.004)	0.203^∗^ (0.102)	0.016^∗∗^ (0.006)	0.544^∗^ (0.231)	0.006	0.075
Non-HDL-C	0.021^∗∗∗^ (0.004)	0.014 (0.095)	0.029^∗∗∗^ (0.006)	0.472^∗^ (0.216)	0.005	0.091
HOMA-β	−1.336^∗^ (0.560)	19.539 (13.761)	3.486^∗∗∗^ (0.795)	−70.717^∗^ (30.583)	0.006	0.061

BMI = body mass index, DBP = diastolic blood pressure, HOMA-β = Homeostasis model assessment of beta cell function, LTL = Leucocyte telomere length, Non-HDL-C = non-high-density lipoprotein cholesterol, SBP = Systolic blood pressure, TC = Total cholesterol. ^∗∗∗^ = *P* < .001 ^∗∗^ = *P* < .01 ^∗^ = *P* < .05.

**Table 4:** Linear regression for the associations of telomere length quartiles with cardio-metabolic variables.

	SBP	DBP	TC	LDL-C	Non-HDL-C	HOMA-β
Age (yr)	0.776^∗∗∗^ (0.077)	0.306^∗∗∗^ (0.051)	0.026^∗∗∗^ (0.004)	0.020^∗∗∗^ (0.004)	0.022^∗∗∗^ (0.004)	−1.326^∗^ (0.560)
Gender (female = reference)	−7.711^∗∗∗^ (1.839)	−2.274 (1.237)	0.222^∗^ (0.102)	0.112 (0.088)	0.030 (0.095)	18.375 (13.781)
Body mass index (kg/m^2^)	0.204 (0.107)	0.242^∗∗^ (0.072)	0.014^∗^ (0.006)	0.024^∗∗∗^ (0.005)	0.028^∗∗∗^ (0.006)	3.521^∗∗∗^ (0.798)
Increasing TL quartile	−4.425^∗^ (1.896)	−2.678^∗^ (1.275)	0.322^∗∗^ (0.106)	0.246^∗∗^ (0.091)	0.278^∗∗^ (0.098)	−37.024^∗∗^ (14.120)
*R*-squared univariate	0.007	0.006	0.003	0.002	0.002	0.008
*R*-squared with confounders	0.17	0.076	0.084	0.094	0.098	0.063

Legend: DBP = diastolic blood pressure, HOMA-β = Homeostasis model assessment of beta cell function, LDL-C = low-density lipoprotein cholesterol, Non-HDL-C = non-high-density lipoprotein cholesterol, SBP = Systolic blood pressure, T C = Total cholesterol. ^∗∗∗^ = *P* < .001 ^∗∗^ = *P* < .01 ^∗^ = *P* < .05.

In the logistic regression with similar levels of adjustments, Log_10_ TL was associated with hypercholesterolaemia and raised WHtR but not with other dyslipidaemia or obesity variables (Table 5). Quartiles of TL were associated with the prevalence of hypertension and dyslipidaemia (hypercholesterolaemia and raised LDL-C and non-HDL-C) (Table 6). TL was not significantly associated with diabetes or obesity defined by BMI, WC, and WHR.

**Table 5:** Logistic regression models (odds ratios and 95% confidence intervals) for the associations of Log_10_ telomere length with cardio-metabolic conditions.

	Age (yr)	Gender (female = reference)	BMI (kg/m^2^)	Log_10_LTL	*c*-statistics
Obesity (WHtR > 0.5)	0.94^∗∗∗^ (0.91–97)	1.61 (0.85–3.06)	0.50^∗∗∗^ (0.44–0.56)	5.67^∗^ (1.14–28.22)	0.65
Hypertension	1.10^∗∗∗^ (1.08–1.12)	1.02 (0.65–1.59)	1.04^∗∗^ (1.02–1.07)	0.44 (0.16–1.23)	0.72
Diabetes	0.93^∗∗∗^ (0.90–0.96)	0.63 (0.31–1.29)	0.94^∗∗^ (0.90–0.98)	0.44 (0.08–2.35)	0.71
Total cholesterol > 5mmol/L	0.96^∗∗∗^ (0.94–0.97)	1.11 (0.69–1.79)	0.98 (0.95–1.00)	0.28^∗^ (0.10–0.81)	0.63
HDL-C < 1.2 mmol/L	1.02^∗^ (1.00–1.04)	0.40 ^∗∗∗^ (0.26–0.61)	0.95^∗∗∗^ (0.93–0.98)	0.94 (0.38–2.56	0.62
Triglycerides > 1.5 mmol/L	0.95^∗∗∗^ (0.93–0.97)	0.37^∗∗∗^ (0.22–0.61)	0.96^∗∗^ (0.92–0.98)	0.94 (0.27–3.24)	0.68
LDL-C > 3 mmol/L	0.96^∗∗∗^ (0.94–0.97)	1.22v0.76–1.97)	0.96^∗∗^ (0.93–0.98)	0.40 (0.14–1.14)	0.64
Non-HDL-C > 3.37 mmol/L	0.95^∗∗∗^ (0.94–0.97)	1.02 (0.65–1.59)	0.96^∗∗∗^ (0.93–0.98)	0.43 (0.16–1.18)	0.64

BMI = body mass index, HDL-C = high-density lipoprotein cholesterol, LDL-C = low-density lipoprotein cholesterol, LTL = Leucocyte Telomere length. ^∗∗∗^ = *P* < .001, ^∗∗^ = *P* < .01, ^∗^ = *P* < .05.

**Table 6:** Logistic regression models (odds ratios and 95% confidence intervals) for the associations of telomere length quartile and cardio-metabolic conditions.

	Obesity (WHtR > 0.5)	Hypertension	Diabetes	Total cholesterol > 5 mmol/L	HDL-C < 1.2 mmol/L	Triglyceride > 1.5 mmol/L	LDL-C > 3 mmol/L	Non-HDL-C > 3.37 mmol/L
Age (years)	0.94^∗∗∗^ (0.91–0.97)	1.09^∗∗∗^ (1.07–1.11)	0.94^∗∗∗^ (0.91–0.96)	1.05^∗∗∗^ (1.03–1.07)	1.03^∗∗^ (1.01–1.05)	1.04^∗∗^ (1.02–1.07)	1.05^∗∗∗^ (1.03–1.067)	0.95^∗∗∗^ (0.94–0.97)
Gender (female = reference	1.57 (0.83–2.98)	0.86 (0.54–1.36)	0.73 (0.35–1.53)	0.93 (0.57–1.52)	0.44^∗∗∗^ (0.28–0.67)	1.96^∗^ (1.15–3.34)	0.80 (0.49–1.31)	1.03 (0.66–1.62)
Body mass index (kg/m^2^)	0.50^∗∗∗^ (0.45–0.57)	1.03^∗^ (1.00–1.06)	0.95^∗^ (0.91–0.99)	1.03 (0.97–1.05)	0.96^∗∗^ (0.93–0.98)	1.03 (0.99–1.06)	1.04^∗∗^ (1.01–1.07)	0.96^∗∗^ (0.93–0.98)
TL 2nd quartile	1.06 (0.51–2.23)	0.93 (0.59–1.47)	0.56 (0.25–1.26)	2.31^∗∗^ (1.38–3.89)	1.54 (0.98–2.41)	1.14 (0.63–2.04)	1.69^∗^ (1.04–2.73)	1.77^∗^ (1.11–2.81)
TL 3rd quartile	0.76 (0.37–1.58)	0.81 (0.51–1.28)	0.48 (0.22–1.26)	2.12^∗∗^ (1.25–3.59)	0.89 (0.58–1.39)	1.25 (0.69–2.27)	1.23 (0.75–2.03)	1.46 (0.91–2.33)
TL 4th quartile	0.64 (0.31–1.32)	0.63^∗^ (0.39–1.01)	0.79 (0.34–1.85)	1.94^∗^ (1.14–3.29)	1.08 (0.69–1.67	1.26 (0.70–2.27)	1.62^∗^ (0.99–2.63)	1.57 (0.98–2.51)
*c*-statistics (TL 4th quartile)	0.96	0.72	0.71	0.65	0.62	0.69	0.64	0.65

HDL-C = high- density lipoprotein cholesterol, LDL-C = low-density lipoprotein cholesterol, TL = Telomere length. ^∗∗∗^ = *P* < .001, ^∗∗^ = *P* < .01, ^∗^ = *P* < .05.

## 4 Discussion

TL shortening was significantly associated with some CMD risk factors in HIV infected participants in this study, even after accounting for the confounding effects of age, gender, and BMI. The significant associations with shorter TL included

1. BP variables and prevalent hypertension,
2. hypercholesterolaemia and levels of TC and LDL-C, and
3. insulin secretion defects (HOMA-β).

Furthermore, elevated ALT, a liver enzyme, was inversely and significantly correlated with shorter TL.

Our results accord with other studies that have shown associations between TL and CMD in diverse populations. TL shortening was associated with hypertension and/or increased systolic and/or diastolic BPs in cross-sectional studies in general populations of Lebanon,^[8]^ China,^[32]^ Taiwan^[33]^ and USA adults,^[34,35]^ and children.^[36]^ The possible mechanism of action is bi-directional: short TL may induce hypertension, while factors that contribute to hypertension may enhance telomere shortening. Short TL may induce endothelial cells and vascular smooth muscle cells dysfunction, and insulin resistance which contribute to the development of raised BP and hypertension.^[32,37]^ On the other hand, oxidative stress and inflammation, which are risk factors for hypertension, also lead to telomere shortening.^[38]^ Although causality and the pathway for TL shortening cannot be established in this cross-sectional study, the significant negative correlation between TL and ALT shown may suggest the influence of oxidative stress on TL shortening. High levels of ALT, a marker of abnormal liver function and indicative of hepatocyte damage, often precede oxidative stress.^[39]^ This suggests that oxidative stress may have contributed to telomere shortening in this study.

The association of short TL with hypercholesterolaemia and rising LDL-C in this study may be via the substantial contribution of altered serum lipids to systemic inflammation and oxidative stress, which in turn induces telomere shortening.^[38]^ Moreover, hypercholesterolaemia is associated with cellular damage, chronic subclinical inflammation, and cell replication, which lead to telomere shortening.^[40]^ Similar findings have been reported in diverse populations in cross-sectional studies from China,^[41]^ Iran^[42]^ and United States.^[34]^

Our study reported a linear inverse association between TL and HOMA-β, which increases with beta cell dysfunction and precedes progression to diabetes.^[43]^ Short telomeres may lead to premature β-cell death, resulting in reduced β-cell mass, impaired insulin secretion,^[44]^ and diabetes. However, no association was found between short TL and diabetes in this study, which may likely be due to the relatively few participants with diabetes (n = 61) in the sample. Nevertheless, the association between short TL and diabetes has consistently been reported in the literature.^[45]^

The association between TL and obesity is controversial as reveal by a systematic review with 38% of the studies showing no association.^[46]^ Most of the studies used BMI to define obesity and all studies with association reported a significant negative association between TL and obesity. In our study, the association between TL and obesity was found only with WHtR > 0.5.^[46]^ Regression analysis adjusting for age and sex showed an association between short TL and WHtR > 0.5. WHtR may be a better predictor for obesity and CMDs such as raised BP, dyslipidaemia and altered glucose metabolism in this population than BMI or WC, which were not associated with short TL.^[47]^ The association of telomere shortening with WHtR, but not WC or BMI, likely add to the body of literature that supports the use of WHtR to identify increased cardiometabolic risk.

Our study did not find any association between TL and age probably because of the narrow age range (median age 38 years, IQR = 32–45). However, TL has been widely reported as a marker of ageing characterised by TL shortening with older age.^[48–52]^ Nevertheless, the cardio-metabolic abnormalities of obesity (WHtR > 0.5), HOMA-β, lipids levels (rising LDL-C and rising TC), higher systolic and diastolic BPs and hypertension were significantly associated with short TL and ageing in our study.

### 4.1 Strength and limitations

The main limitation of this study is the cross-sectional design which prevents inferring causal associations between TL and CMD risk profile. Moreover, the absence of a control group precludes controlling for the effects of HIV and ART which influence both TL and CMDs. The underlying mechanisms are only hypothesised since markers of oxidative stress and inflammation, and telomerase activity were not measured. Considering that most HIV-related studies in Africa are single-clinic-based, a major strength of this study is the inclusion of multiple healthcare facilities. Moreover, the included healthcare facilities, selected using random sampling methods, were based in both urban and rural areas, which allows for the generalizability of the results to other South African HIV-infected populations.

Including a control group in future studies to control for the effect of HIV and ART is essential. The direction of the association, if established in longitudinal studies, will enable the use of TL as a possible biomarker for the early identification hypertension, dyslipidaemia and diabetes in PLWH on ART in South Africa.

## 5 Conclusions

The association of TL shortening with hypertension, dyslipidaemia and defects in insulin secretion in PLWH on ART in South Africa suggests that TL may be used as an early biomarker of these CMDs in this population. More research is needed to explore the direction of these associations in longitudinal studies, and to examine differences in TL shortening in HIV-infected ART-naïve and HIV-uninfected populations with CMDs. Such data will provide greater insights on the contributors to TL shortening and may perhaps lead to new therapies in future.

## Author contributions

**Conceptualization:** Tandi E. Matsha, Andre P. Kengne.

**Formal analysis:** Andre P. Kengne.

**Funding acquisition:** Andre P. Kengne.

**Investigation:** Tandi E. Matsha, Andre P. Kengne.

**Project administration:** Tandi E. Matsha, Andre P. Kengne.

**Supervision:** Nasheeta Peer, Tandi E. Matsha, Anniza de Villiers, Eugene Sobngwi, Andre P. Kengne.

**Validation:** Nasheeta Peer, Tandi E. Matsha, Andre P. Kengne.

**Writing – original draft:** Ndonwi Elvis Ngwa.

**Writing – review & editing:** Ndonwi Elvis Ngwa, Nasheeta Peer, Anniza de Villiers, Eugene Sobngwi, Andre P. Kengne.
